# Long-term changes in gene expression in fetal alcohol spectrum disorders

**Published:** 2013-07

Consumption of alcohol during pregnancy is associated with a variety of birth defects. Alcohol-related birth defects are collectively known as fetal alcohol spectrum disorders (FASDs), which are a leading cause of cognitive defects in North America. Studies using animal models have shown that alcohol induces global changes in gene expression in the developing brain. Using an FASD mouse model that they previously established, Shiva Singh and colleagues tested the hypothesis that long-term alterations in gene expression, mediated by epigenetic mechanisms, are a feature of FASDs. Developing mice were exposed to alcohol and, as adults, their epigenomes were assessed for changes in DNA methylation patterns and non-coding RNA (ncRNA) expression. The analysis unveiled long-lasting alterations in DNA methylation in response to fetal alcohol exposure. These changes mapped to the promoters of certain ncRNAs, implicating ncRNA deregulation in FASDs. **Page 977**

